# Extrusion-Based 3D Printing of Poly(ethylene glycol) Diacrylate Hydrogels Containing Positively and Negatively Charged Groups

**DOI:** 10.3390/gels4030069

**Published:** 2018-08-14

**Authors:** Sebastian Joas, Günter E. M. Tovar, Oguz Celik, Christian Bonten, Alexander Southan

**Affiliations:** 1 Institute of Interfacial Process Engineering and Plasma Technology IGVP, University of Stuttgart, Nobelstr. 12, 70569 Stuttgart, Germany; sebastian.joas@gmx.net (S.J.); guenter.tovar@igvp.uni-stuttgart.de (G.E.M.T.); 2 Institut für Kunststofftechnik IKT, University of Stuttgart, Pfaffenwaldring 32, 70569 Stuttgart, Germany; oguz.celik@ikt.uni-stuttgart.de (O.C.); christian.bonten@ikt.uni-stuttgart.de (C.B.); 3 Fraunhofer Institute for Interfacial Engineering and Biotechnology IGB, Nobelstr. 12, 70569 Stuttgart, Germany; Guenter.Tovar@igb.fraunhofer.de

**Keywords:** 3D printing, hydrogels, polyelectrolyte, shear thinning, yield stress, viscosity, rheology, FT-IR spectroscopy

## Abstract

Hydrogels are an interesting class of materials used in extrusion-based 3D printing, e.g., for drug delivery or tissue engineering. However, new hydrogel formulations for 3D printing as well as a detailed understanding of crucial formulation properties for 3D printing are needed. In this contribution, hydrogels based on poly(ethylene glycol) diacrylate (PEG-DA) and the charged monomers 3-sulfopropyl acrylate and [2-(acryloyloxy)ethyl]trimethylammonium chloride are formulated for 3D printing, together with Poloxamer 407 (P407). Chemical curing of formulations with PEG-DA and up to 5% (*w/w*) of the charged monomers was possible without difficulty. Through careful examination of the rheological properties of the non-cured formulations, it was found that flow properties of formulations with a high P407 concentration of 22.5% (*w/w*) possessed yield stresses well above 100 Pa together with pronounced shear thinning behavior. Thus, those formulations could be processed by 3D printing, as demonstrated by the generation of pyramidal objects. Modelling of the flow profile during 3D printing suggests that a plug-like laminar flow is prevalent inside the printer capillary. Under such circumstances, fast recovery of a high vicosity after material deposition might not be necessary to guarantee shape fidelity because the majority of the 3D printed volume does not face any relevant shear stress during printing.

## 1. Introduction

The formulation of hydrogels for extrusion-based 3D printing has attracted increasing attention in recent publications [1,2,3]. This is, on the one hand, based on the favorable properties of hydrogels in general, namely, their viscoelastic properties [4,5], their high water content [6] and their often low toxicity [7,8,9]. On the other hand, extrusion-based 3D printing offers a relatively quick way to generate 3D structured hydrogel objects in the centimeter range without the need for molds [10,11,12], as used, e.g., in injection molding [13]. Looking at materials used for hydrogel formulations in general, both bio-based polymers like hyaluronic acid [14,15], gelatin [16,17], and alginate [18,19] as well as synthetic polymers like polyacrylamide (PAAm) [20], polyvinyl alcohol (PVA) [21], and poly(ethylene glycol) (PEG) [22,23] have been used in various applications and were partly formulated for extrusion-based 3D printing as well [24,25,26]. In particular, the readily available PEG can be obtained in various polymer architectures and with various end-group functionalities. This facilitates the formation of PEG-based hydrogels through different cross-linking reactions, such as radical polymerization [27] or the thiol–Michael reaction [28] involving acrylate end groups, thiol-ene reactions involving unsaturated end groups [29], or enzymatic cross-linking using lysine and glutamine end groups [30]. In all of these cases, it is possible to introduce functional building blocks into the otherwise inert PEG hydrogel, such as oligopeptides [31,32], charged moieties [33], or degradable groups [34]. Adding functional building blocks is particularly easy in the case of radically cross-linkable poly(ethylene glycol) diacrylate (PEG-DA). Many functional acrylate monomers are commercially available and can be integrated into the hydrogel network simply by mixing the monomers into the hydrogel formulation before curing [35]. In this way, charged monomers were also integrated into PEG-DA hydrogels, resulting in tuned properties concerning, e.g., the equilibrium degree of swelling (*EDS*), stiffness, mineralization of inorganic salts inside the hydrogels, and the response of biological cells [33,36,37]. However, many of the hydrogel formulations reported before the advent of additive manufacturing techniques do not have properties that fit into the processing window, thus making their reformulation mandatory with respect to the processing technique used. Compared to other additive manufacturing techniques for hydrogel processing, like inkjet printing [17,38], extrusion-based 3D printing usually uses highly viscous physical hydrogels which are squeezed through a nozzle with a diameter that is typically not smaller than 200 μm [39]. Suitable formulations should exhibit shear thinning behavior, and the yield stress is also recognized as an important property [24,40]. For extrusion-based 3D printing of PEG-DA, the thermoresponsive block copolymer Poloxamer 407 (P407) can be added to the hydrogel formulations, resulting in ideal rheological properties without disturbing the chemical curing of PEG-DA [25,41]. Whereas the yield stress is a key factor in the achievable object height [42], shear thinning of the formulations has a direct impact on the shear rate profile in the 3D printing nozzle, resulting in a plug-like flow and thus supporting the generation of smooth hydrogel strands during printing [25,43]. However, it can be expected that for formulations possessing significant yield stress in combination with shear thinning behavior, an interaction between yield stress and shear thinning concerning the flow profile becomes effective, as already shown for injectable hydrogels [44,45,46]. Motivated by the effect of charged groups on the properties of PEG-based hydrogels as described above, in this contribution, PEG-based hydrogel formulations are developed which contain positively or negatively charged groups and exhibit the crucial rheological properties for extrusion-based 3D printing. Both the physical properties of non-cured and cured formulations are investigated in detail. In particular, the rheological properties are assessed and the impacts of the yield stress and shear thinning behavior on the flow profile in the 3D printing nozzle are examined. Thus, we aim to expand the material platform of PEG-DA-based hydrogels for extrusion-based 3D printing as well as to contribute to understanding the impact of rheological properties on the 3D printability of hydrogel formulations in general.

## 2. Results

### 2.1. Physical Properties of Cross-Linked Hydrogels

**Figure 1:**
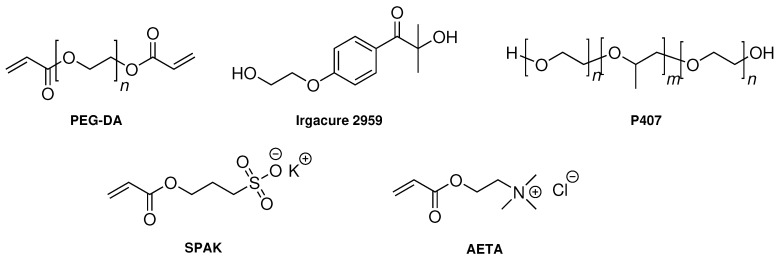
Structures of poly(ethylene glycol) diacrylate (PEG-DA), Irgacure 2959, Poloxamer 407 (P407), sulfopropyl acrylate potassium salt (SPAK), and [2-(acryloyloxy)ethyl]trimethylammonium chloride (AETA).

The hydrogel formulations in this study contained P407, PEG-DA, the photo-initiator Irgacure 2959, and one of two charged monomers—sulfopropyl acrylate potassium salt (SPAK) or [2-(acryloyloxy)ethyl]trimethylammonium chloride (AETA) (see Figure 1). P407 was added to induce physical gelation in order to reach properties which fit into the processing window of extrusion-based 3D printing [47]. On the other hand, PEG-DA, the charged monomers, and Irgacure 2959 were present to facilitate chemical curing by UV irradiation.

In the context of extrusion-based 3D printing, effective curing after printing is particularly important in order to fix the shape of a printed object permanently. Therefore, the first step was to evaluate whether the PEG-DA end groups as well as the acrylic functions of the charged monomers SPAK and AETA could be photo-polymerized successfully to form hydrogels in the presence of P407. For this purpose, solutions with different concentrations of SPAK and AETA, respectively, were prepared and irradiated with UV light. The compositions of the hydrogel precursor solutions are given in Table 1 and Table 2.

**Figure 2:**
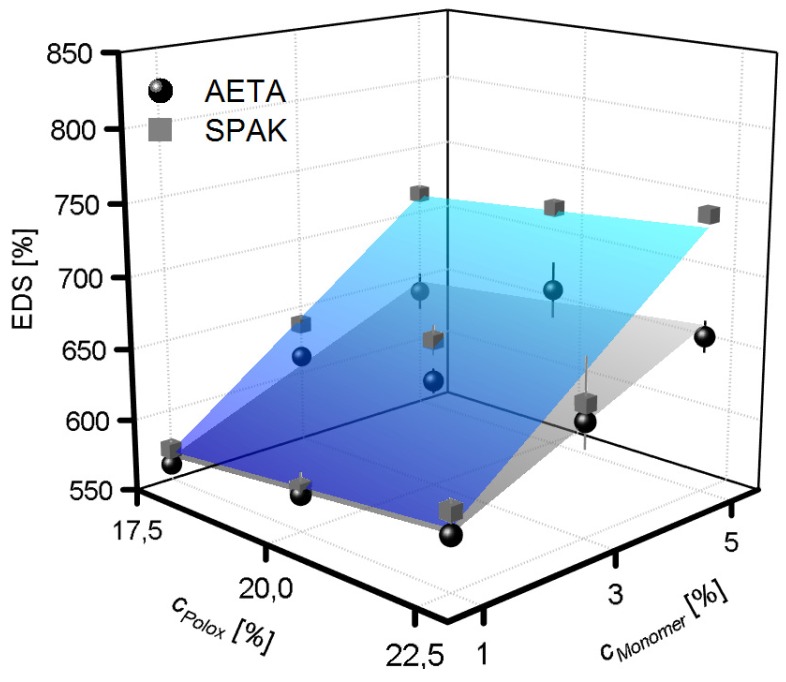
Equilibrium degree of swelling (EDS) of cross-linked hydrogels with varying concentrations ($c_{Monomer}$) of positively and negatively charged monomers. The surface fits to the data points are to guide the eye only.

Generally, upon UV curing, solid discs were obtained from all hydrogel formulations tested. Whereas the non-cured formulations were dissolved upon adding water, the cured formulations formed stable hydrogels which swelled only up to a certain equilibrium water content. Swelling was characterized by measuring the equilibrium degree of swelling (EDS) of the cured formulations (Figure 2). The EDS values ranged between 561% and 665% for the AETA-containing hydrogels and between 572% and 743% for the SPAK-containing hydrogels. The smallest EDS was found in both cases with the lowest $c_{Polox}$ of 17.5% and the lowest concentration of the charged monomer of 1%. The EDS increased slightly with $c_{Polox}$; however this effect was rather small. A larger effect was caused by an increase in the charged monomer concentration, also resulting in increased EDS values. Comparing SPAK and AETA, the addition of SPAK resulted in a larger EDS increase compared to AETA.

**Figure 3:**
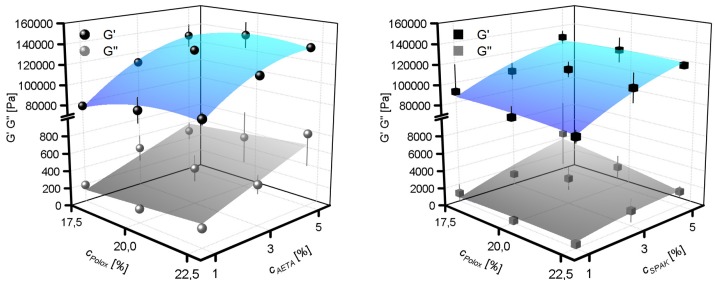
Storage moduli *G*′ and loss moduli *G*″ of cross-linked hydrogels with varying concentrations of $c_{AETA}$ (positively charged monomers; **left**) and $c_{SPAK}$ (negatively charged monomers; **right**). The surface fits to the data points are to guide the eye only.

Additionally, mechanical hydrogel properties were assesed by measuring the storage moduli *G*′ and loss moduli *G*″ of the cured hydrogels (Figure 3) for both AETA-containing hydrogels and SPAK-containing hydrogels. Values for *G*′ ranged between 76 kPa and 139 kPa for AETA-containing hydrogels and between 79 kPa and 127 kPa for SPAK-containing hydrogels. *G*″ ranged between 0.2 kPa and 0.8 kPa for AETA-containing hydrogels and between 0.3 Pa and 4.5 kPa for SPAK-containing hydrogels. It was evident for all formulations tested with charged monomer concentrations between 1% and 5% that after curing the *G*′ values were approximately two orders of magnitude larger than the *G*″ values. This observation is in contrast to the mechanical properties of the formulations before curing, where *G*′ and *G*″ in the physical gel state (see results in the following section) were generally of comparable magnitudes (Supplementary Figure S1). Similar to the EDS, the *G*′ and *G*″ of the cured hydrogels were hardly influenced by $c_{Polox}$, whereas the addition of one of the two charged monomers resulted in an increase of both *G*′ and *G*″. However, hardly any differences in mechanical properties between the SPAK- and AETA-containing hydrogels were found.

**Figure 4:**
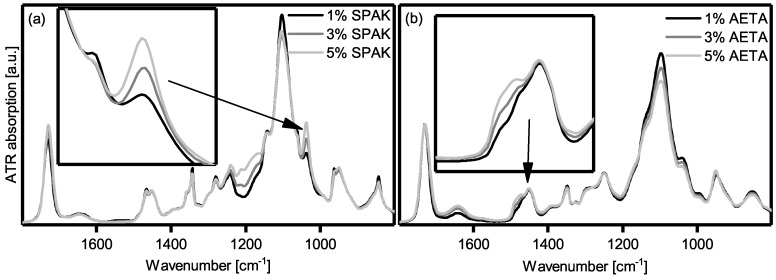
ATR-FT-IR spectra of hydrogels containing 22.5% Poloxamer 407 and different amounts of (**a**) SPAK and (**b**) AETA, respectively. The insets show the respective characteristic absorption bands for the charged monomers.

In order to investigate further if the charged monomers were incorporated into the hydrogel polymer network, the dry masses of the hydrogels were determined after washing and swelling and were generally very close to the mass of the starting materials used. Additionally, the FT-IR spectra of dried hydrogel samples were recorded (Figure 4). For the SPAK-containing hydrogels, an absorption band with increasing intensity parallel to $c_{SPAK}$ was present at 1040 cm^−1^. Also, for AETA containing hydrogels, a shoulder was found at around 1470 cm^−1^, increasing in intensity with $c_{AETA}$.

Additionally, the supernatant waters were analyzed after washing of the hydrogels. By drying and subsequent ^1^H NMR spectroscopy as well as size exclusion chromatography (SEC), the solid white residue was identified as unmodified P407. The total mass of recovered P407 from the hydrogels by washing was practically quantitative (Supplementary Figure S2).

### 2.2. Physical Gelation of Hydrogel Formulations with Charged Monomers

**Figure 5:**
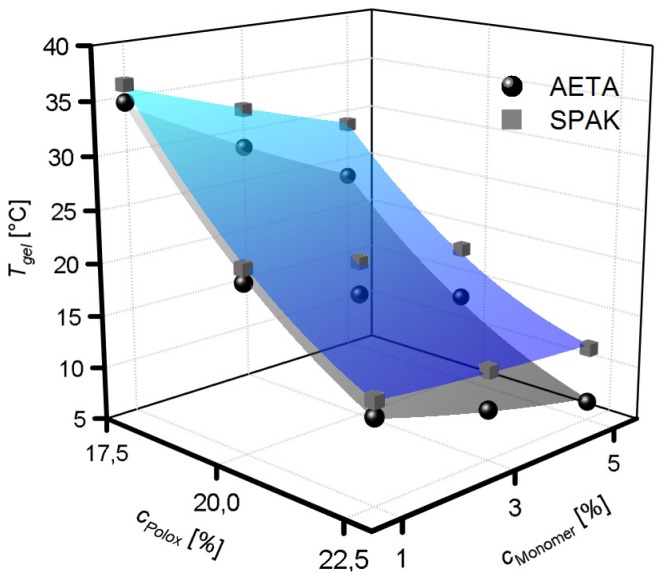
Gel transition temperatures ($T_{gel}$) of ink formulations with varying concentrations ($c_{Monomer}$) of positively and negatively charged monomers. The surface fits to the data points are to guide the eye only.

Chemical curing of the formulations as described above becomes important after completion of a 3D printed object. However, before curing becomes relevant, the formulations have to flow through the 3D printer nozzle and solidify rapidly after deposition on the collector platform. In case of the formulations tested here, this should be achieved by the addition of P407 in such concentrations that physical hydrogels are formed by P407 micelle entanglement. Therefore, it is crucial to measure if the physical gelation of P407 is hampered in any way by adding SPAK or AETA. The $T_{gel}$ values of hydrogel formulations containing SPAK and AETA are shown in Figure 5. In all formulations tested with charged monomer concentrations between 1% and 5%, a $T_{gel}$ was measured and accordingly, physical hydrogels were obtained above the individual $T_{gel}$ values. The rheological measurements are shown in the Supplementary Figure S3. $T_{gel}$ values decreased with increasing $c_{Polox}$. As a general trend, $T_{gel}$ values decreased upon the addition of charged monomers, although this effect was slightly different for SPAK and AETA, and also, an interaction with $c_{Polox}$ was observed. At a low $c_{Polox}$ of 17.5%, the addition of SPAK to the formulations led to $T_{gel}$ values of 36.1 ${}^{\circ }$C (1% SPAK) and 28.6 ${}^{\circ }$C (5% SPAK), whereas the addition of AETA led to $T_{gel}$ values of 34.5 ${}^{\circ }$C (1% AETA) and 23.1 ${}^{\circ }$C (5% AETA). In contrast, at high $c_{Polox}$ of 22.5%, $T_{gel}$ values ranged between 14.3 ${}^{\circ }$C (1% SPAK), 12.0 ${}^{\circ }$C (5% SPAK), 12.8 ${}^{\circ }$C (1% AETA), and 6.8 ${}^{\circ }$C (5% AETA). Thus, at low $c_{Polox}$, a decrease in $T_{gel}$ values of 7.5 ${}^{\circ }$C (SPAK) and 11.4 ${}^{\circ }$C (AETA) were observed, whereas at high $c_{Polox}$, decreases of only 2.3 ${}^{\circ }$C (SPAK) and 6.0 ${}^{\circ }$C (AETA) were measured. Interestingly, the *G*′ and *G*″ values of the physical hydrogels measured 10 ${}^{\circ }$C above $T_{gel}$ depended only on $c_{Polox}$ and not on $c_{SPAK}$, whereas $c_{AETA}$ had a significant effect on *G*′ and *G*″ (Supplementary Figure S1).

### 2.3. Flow Properties of Hydrogel Formulations with Charged Monomers

**Figure 6:**
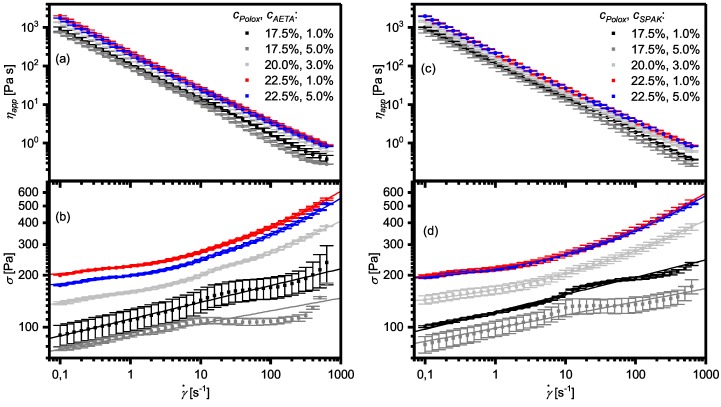
(**a**) Apparent viscosity $\eta_{app}$ and (**b**) shear stress σ against shear rate $\dot{\gamma }$ of hydrogel formulations containing AETA measured at temperatures 10 K above $T_{gel}$. (**c**) The apparent viscosity ($\eta_{app}$) and (**d**) the shear stress (σ) versus the shear rate ($\dot{\gamma }$) of hydrogel formulations containing SPAK measured at temperatures 10 K above $T_{gel}$. In (**b**,**d**), data belonging to formulations with $c_{Polox}$ ≥ 20% were fitted using Equation (3), and formulations with $c_{Polox}$ = 17.5% were fitted using Equation (1) (for equations see Section 4.2).

Apart from the ability to form physical hydrogels, a key feature of hydrogel formulations for extrusion-based 3D printing is their flow behavior when shear forces are applied, like during 3D printing. Therefore, we aimed to characterize the flow properties of our hydrogel formulations in terms of shear thinning behavior and yield stress. For this purpose, we measured the shear stress (σ) and apparent viscosity $\eta_{app}$ against the shear rate $\dot{\gamma }$. The results are shown in Figure 6. Looking at the viscosities (Figure 6a,c), the viscosity curves were straight lines in the double logarithmic plot, indicating that they can be fitted well with a power law in the form of Equation (2) (Section 4.2). However, the viscosity plots conceal that a power law was not appropriate to fit all data curves. This becomes especially clear when looking at the shear stress against the shear rate obtained from the same measurements (Figure 6b,d). According to Equation (1) (Section 4.2), these curves should also be straight lines in a double logarithmic plot which is not the case, especially for formulations with $c_{Polox}$ ≥ 20%. In these cases, higher shear stresses at lower shear rates than expected by a power law were observed. Such behavior can be described better with a Herschel–Bulkley model using Equation (3) (Section 4.2). The resulting fits show an excellent agreement with the experimental data and indicate that the formulations with higher $c_{Polox}$ possess a yield stress. The formulations with $c_{Polox}$ = 17.5% can still be fitted best using a power law which indicate that they possess only a very small or no yield stress. However, their flow behavior tends to be more complex, especially at higher shear rates, so that also the power law can only give an estimation of the flow behavior of these formulations. The results of the fit parameters $K_{PL}$ and *n* for formulations with $c_{Polox}$ = 17.5% are shown in Table 1. The fit parameters *m*, $K_{HB}$, and $\sigma_{y}$ obtained for formulations with $c_{Polox}$ ≥ 20% are listed in Table 2. Values for *n* were in the range of 0.1, and for *m*, they were around 0.25, evidencing that all formulations showed pronounced shear thinning behavior.

### 2.4. Influence of Yield Stress and Shear Thinning on Flow Profiles

**Figure 7:**
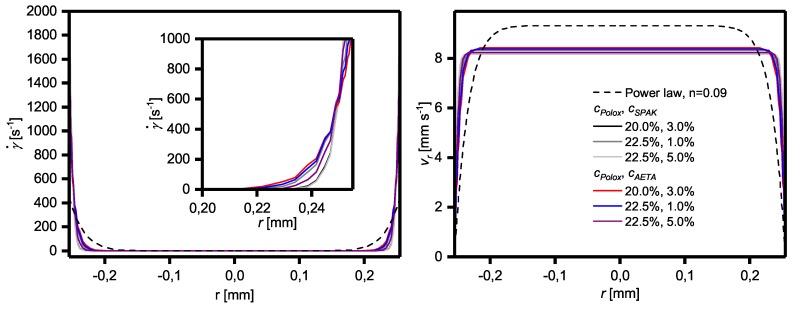
Calculated shear rate and velocity profiles of a power law liquid with *n* = 0.09 (dashed line), representative of formulations containing 17.5% Poloxamer 407, and of the different Herschel–Bulkley liquids from Table 2 which have a $c_{Polox}$ ≥ 20%.

Because the flow properties of the solutions have a pronounced influence on the flow profile of the formulations during extrusion-based 3D printing experiments and thus, on the resulting fabricated objects, the flow profiles of the formulations in terms of shear rate and flow velocity were calculated both using a power law model according to Kraut et al. [25] and the Herschel–Bulkley model. The results are shown in Figure 7. The flow profile perpendicular to the flow direction was independent of the position along the flow direction, showing that the flow remains laminar under 3D printing conditions.

Looking at the shear rate profile of the formulations with $c_{Polox}$ ≥ 20% obeying a Herschel–Bulkley model, it is evident that relevant shear rates only occur very near to the dispensing needle wall. Due to the yield stress, this plug-like flow is even more pronounced than that compared to the formulations with $c_{Polox}$ = 17.5% which can also be described with a power law. Thus, for the Herschel–Bulkley liquids, the maximum shear rates at the wall are much higher than for the power law fluids which amount to values of up to 2405 s^−1^ and 442 s^−1^, respectively. The corresponding flow velocities in the center of the dispensing needle (r = 0) are therefore very close to the average flow velocity of 8 mm s^−1^. As a consequence, the Herschel–Bulkley fluids face hardly any shear stress during 3D printing in the majority of the printed volume, as also shown in the Supplementary Figure S4.

### 2.5. Viscosity Recovery after Shearing

Knowing the recovery kinetics of formulation viscosity after shearing is important for assessing the suitability of the formulation for extrusion-based 3D printing. Therefore, the viscosities of the formulations were measured after a sudden increase to 300 s^−1^ or decrease to 0.01 s^−1^, respectively, of the shear rate (Supplementary Figures S5 and S6). It was found that when applying a sudden increase in shear rate to the formulations, in all cases, the viscosities dropped instantaneously to a low and constant value. On the other hand, the sudden reduction of shear stress to a very low level, as done when depositing the material on the collector platform, caused the viscosities to increase spontaneously within a few seconds to values close to the viscosities before shearing.

### 2.6. Extrusion-Based 3D Printing of Hydrogel Formulations

**Figure 8:**
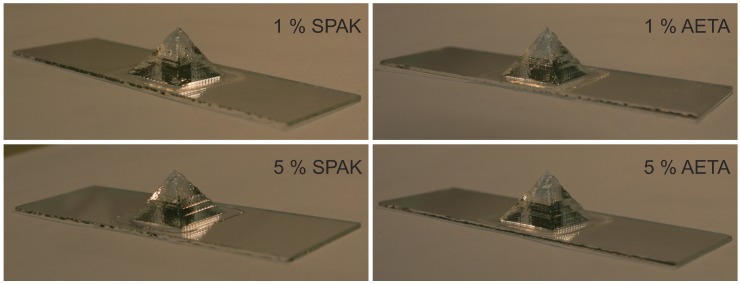
Representative photos of 3D printed hydrogel pyramids of formulations containing 1% and 5% of SPAK and AETA, respectively, as well as 22.5% P407. The objects had an edge length of 20 mm and were printed on glass slides with an area of 26 mm × 76 mm.

Three-dimensional printing of the formulations with a $c_{Polox}$ of 22.5% at room temperature proceeded without any noticeable difficulties, and 3D objects of several centimeters in size could be generated. Exemplarily, the 3D printing results of hydrogel pyramids with a base edge length of 2 cm are shown in Figure 8 for formulations containing 1% or 5% of SPAK or AETA, respectively. Their shape reflects the targeted shape defined by the underlying CAD drawing. The non-cured 3D printed objects did not show any sign of material flow over 24 h, indicating that the gravitational stress did not exceed the yield stress of the formulations, and no intermediate curing was necessary during printing [42]. Because our 3D printing setup did not allow heating of the formulations during 3D printing, it was not possible to print formulations containing 17.5% P407.

## 3. Discussion

### 3.1. Physical Properties of Cross-Linked Hydrogels

The observed increase in EDS with the concentrations of the two charged monomers, SPAK and AETA, is the expected behavior for a higher charge density in polyelectrolyte hydrogels due to the high water binding capacity of the ionic groups [48,49]. In fact, all reported EDS values were larger than those for similar PEG-DA hydrogels with $c_{PEG-DA}$ = 20%, $c_{Polox}$ between 17.5% and 25% and an EDS around 520% [41]. The difference in EDS between SPAK and AETA containing hydrogels can be understood as a result of the different chemical structures, e.g., the alkyl chain length between the acrylate and ionic group or the ionic group itself [50,51]. Also, differences in copolymerization parameters between AETA and SPAK with PEG-DA might result in different network architectures and thus changed EDS values. In any case, the EDS data together with the practically quantitative gel yields suggest that chemical cross-linking by UV irradiation was successful and also that the charged monomers were incorporated into the polymer networks.

The change in the ratio of *G*′ and *G*″ for all formulations upon curing also points into the same direction, as it shows a general change from viscous to elastic for all tested formulations upon curing. This behavior is typical for the generation of a three-dimensionally cross-linked polymer network [52].

The conclusion of complete cross-linking was further evidenced by the FT-IR spectra. The new absorption band in SPAK hydrogels can be assigned to the S–O stretching vibration of the sulfonate group [53]. The new absorption in AETA hydrogels is assigned to a C–H bending vibration. The C–N stretching vibration of AETA around 1240 cm^−1^ was not visible, probably due to the presence of more intense absorptions in this region [48]. Because the hydrogels were washed excessively before drying, it can be assumed that monomers that were not polymerized into the hydrogel polymer network were removed and that only covalently bound SPAK and AETA were present.

The quanitative recovery of P407 from the cured hydrogels upon washing with water shows that P407 did not react during radical cross-linking and also did not disturb the cross-linking process in any other noticeable way, as could be expected by the absence of reactive groups in P407. Therefore, the chemical hydrogels obtained after washing consisted only of cross-linked PEG-DA together with a defined amount of one of the two charged monomers. This finding is especially important in view of possible applications in contact with biological systems, because it is known that P407 in high concentrations shows cytotoxic effects [54].

In summary, the formulations allowed the preparation of chemically cross-linked PEG-based hydrogels by photo-polymerization with defined concentrations of the monomers SPAK or AETA, respectively. In the context of extrusion-based 3D printing, the compositions of the formulations, therefore, can be used to tailor the properties of cured 3D printed hydrogel objects.

### 3.2. Physical Gelation of Hydrogel Formulations with Charged Monomers

The physical gelation of P407 is based on micelle formation and entanglement and typically becomes effective above a certain gel transition temperature ($T_{gel}$) [55]. Physical gelation can be monitored by temperature-dependent rheology and is characterized by a sharp increase in *G*′. It is known that the micelle formation and thus, $T_{gel}$ is susceptible to the addition of organic and charged compounds into the solutions, which may ultimately lead to failure of physical gel formation [56]. However, physical gelation is a prerequisite for successful extrusion-based 3D printing of the hydrogel formulations in order to guarantee shape fidelity. It was shown previously that the addition of PEG-DA and Irgacure 2959 is possible while preserving the ability of P407 to form physical gels [41]. However, the presence of SPAK or AETA, respectively, in the formulations makes the situation more complex, and as described above, both SPAK and AETA have an influence on $T_{gel}$.

The observed trend for the general decrease of $T_{gel}$ upon the addition of charged monomers is in accordance with previous findings that the addition of salts decreases the $T_{gel}$ of P407 solutions. This is commonly explained by the high affinity of water molecules to the charged moieties and the resulting reduced hydrogen bond donating ability of the involved water molecules [57,58]. However, the reason for the different effects of SPAK and AETA addition and also the origin of the two-factor interaction between $c_{Polox}$ on the one hand and $c_{AETA}$ and $c_{SPAK}$, respectively, on the other hand cannot be clarified at this point. Generally, in the presence of both charged monomers, physical hydrogels are formed, and thus, P407 micelle formation is still effective. Accordingly, in good agreement with previously published data on Poloxamer 407 hydrogels [57], $T_{gel}$ decreased with increasing $c_{Polox}$ due to more effective entanglement with the Poloxamer 407 micelles at higher concentrations.

Due to their ability to form physical hydrogels, generally, all formulations are potential candidates for 3D printing based on the physical gelation alone. However, at low $c_{Polox}$, the formulations need to be heated above their individual $T_{gel}$ which is above room temperature. Our 3D printed setup did not allow heating of the formulations during printing. Therefore, in the 3D printing experiments described in this contribution, only formulations with the highest $c_{Polox}$ were used with $T_{gel}$ values well below room temperature.

### 3.3. Yield Stress, Shear Thinning, Viscosity Recovery after Shearing, and 3D Printing of Hydrogel Formulations with Charged Monomers

The process of extrusion-based 3D printing can be divided into three phases. In the first phase, the hydrogel formulation is stored in a reservoir container with a relatively large cross-section. Therefore, the formulation is usually not sheared to a relevant extent, even during printing. However, in the second phase, the formulation approaches the printer nozzle during printing and the cross-section suddenly decreases. Then, the formulation suddenly experiences large shear forces. In the third phase, the formulation is released from the nozzle and deposited onto a build platform where, suddenly, shear forces are absent again. In this context, during the transition from the first to the second phase, it is generally accepted that shear thinning of physical hydrogels helps with the deposition of of smooth hydrogel strands on the build platform [3,25]. Also, when entering phase three, the formulations need to solidify rapidly in order to guarantee the shape fidelity of the printed object. Finally, during the build up of 3D structures, a certain yield stress is helpful because the 3D printed objects have to support themselves during 3D printing before a potential final curing step [42,59].

Concerning shear thinning, all investigated formulations showed promising results and therefore are probably suited for extrusion-based 3D printing. Also, the rapid viscosity recovery after shearing supports this conclusion. Formulations based on P407 can be considered to be the gold standard regarding these properties, making them popular as model formulations in extrusion-based 3D printing, e.g., for assessing shape fidelity [60].

However, the formulations here also showed differences in yield stress. These apparent differences in yield stress between formulations with smaller and larger $c_{Polox}$ is generally in accordance with literature reports on P407 solutions [47]. At the lower concentrations, entanglements between the P407 micelle coronas are presumably too weak to effect a yield stress.

As shown above, the yield stress also affects the flow of the formulations through the dispensing needle. With a high yield stress, a highly sheared region exists very near to the needle wall which forms a lubricating layer for the formation of smooth strands. Concerning the optimum properties of a hydrogel formulation for extrusion-based 3D printing, it can therefore be concluded that a relatively high yield stress in combination with pronounced shear thinning above this yield stress is favorable. These findings are generally in accordance with reports given on injectable hydrogels where a high yield stress of hydrogel formulations is, for example, helpful for the survival of biological cells present in the hydrogel due to the low shear stress in most of the volume [44,45,46].

The combination of shear thinning and high yield stress might even have an additional effect. The fact that, for the formulations investigated in this study, only a small volume fraction was sheared to a relevant extent is not only beneficial for smooth strand formation, but also means that after deposition onto the collector platform, most of the material never left its physical gel form during the entire printing process. Thus, the shape fidelity of printed objects should not be impaired significantly due to shear thinning, e.g., by depositing material in a liquid state which needs time to recover to a high viscosity suitable to support the printed structure. Under these circumstances—although a quick viscosity recovery is certainly beneficial for shape fidelity—the longer time in which the surface of the printed strands is in a rather liquid state might facilitate mixing of the surface layers of adjacent strands and thus, improve the connectivity between strands and layers, presumably resulting in improved mechanical properties. However, no experimental data on this assumption are available up to now which might be generated in future studies.

The 3D printing results shown above with $c_{Polox}$ = 22.5% demonstrate that the rheological characteristics of the formulations, in fact, led to 3D printed objects with defined shapes. With lower $c_{Polox}$, no 3D printing experiments were conducted, because our printer setup did not allow defined heating during printing. However, due to the very low to absent yield stress of formulations with a $c_{Polox}$ of 17.5%, it can be assumed that objects formed by these formulations will tend to flow while a 3D printed object is assembled, making intermediate curing necessary. Further 3D printing studies with other physical shear thinning hydrogels without a yield stress will be necessary to gain further insight into this point.

## 4. Materials and Methods

### 4.1. Chemicals

Poloxamer 407 (P407), poly(ethylene glycol) diacrylate (PEG-DA, $M_{n}$ = 700 g mol^−1^), 3-sulfopropyl acrylate potassium salt (SPAK), and [2-(acryloyloxy)ethyl]trimethylammonium chloride (AETA, 80% solution in H_2_O) were obtained from Sigma-Aldrich (St. Louis, MO, USA). The radical photoinitiator 1-[4-(2-hydroxyethoxy)phenyl]-2-hydroxy-2-methyl-1-propan-1-one (Irgacure 2959) was a kind gift from Bodo Möller Chemie GmbH (Offenbach am Main, Germany). All chemicals were used as received without further purification. Water was withdrawn from a Barnstead GenPure xCAD water purification system (Thermo Scientific, Schwerte, Germany).

### 4.2. Analytical Methods

^1^H NMR spectra (500 MHz) were recorded on an Avance 500 spectrometer (Bruker, Billerica, MA, USA) with chloroform-*d_1_* as the solvent. Size exclusion chromatography (SEC) measurements in tetrahydrofuran (THF) with a polystyrene calibration were performed at 40 ${}^{\circ }$C on a 1260 Infinity GPC-SEC Analysis System (Agilent Technologies, Santa Clara, CA, USA) equipped with a refractive index (RI) detector and a column combination of two PSS SDV lin S (PSS Polymer Standards Service GmbH, Mainz, Germany) columns. ATR-FT-IR spectra were recorded on a Bruker Equinox 55 spectrometer. Freeze-drying was performed using an Alpha 1-2 LD device (Martin Christ Gefriertrocknungsanlagen GmbH, Osterode am Harz, Germany). UV irradiation was carried out in a UV chamber Sol2 (Dr. Hönle AG, Gräfelfing, Germany). All rheological measurements were performed on a Physica MCR 301 (Anton Paar, Graz, Austria). For the measurement of *G*′ and *G″* of the swollen hydrogels, oscillatory rheology was performed using a parallel plate geometry with a diameter of 20 mm. Amplitude sweeps (frequency = 1 Hz, amplitudes between 0.01% and 10%) and frequency sweeps (amplitude = 0.1 %, frequencies between 0.1 Hz and 100 Hz) for the swollen chemical hydrogels were obtained with a normal force of 1 N at a temperature of 20 ${}^{\circ }$C. For the measurement of *T_gel_* of the non-cured hydrogel formulations, temperature dependent oscillatory rheology between 1 ${}^{\circ }$C and 50 ${}^{\circ }$C was performed using a parallel plate geometry with a diameter of 40 mm with a constant frequency of 1 Hz, a constant amplitude of 5%, and a temperature ramp of 1 ${}^{\circ }$C min^−1^. *T_gel_* was determined from the temperature sweeps as the maximum of the first derivative of *G*′. Flow curves (apparent viscosity ηapp against shear rate $\dot{\gamma }$) of the non-cured formulations were measured using a coaxial cylinder geometry with a logarithmic shear rate ramp between 0.1 s^−1^ and 1000 s^−1^. The viscosity recovery after shearing was also measured with a coaxial cylinder geometry at a constant shear rate of 0.01 s^−1^ for 60 s which was increased suddenly to 300 s^−1^ and kept at this value for for 60 s, followed by three cycles of low and high shear rates. Flow curves were assessed using an empirical power law in accordance with Kraut et al. [25] (Equation (2)) and the shear stresses σ against $\dot{\gamma }$ were either fitted with a power law (Equation (1)) or with the Herschel–Bulkley model (Equation (3)):

(1) $$\sigma =K_{PL}\cdot {\dot{\gamma }}^{n}$$

(2) $$\eta_{app}=\frac{\sigma }{\dot{\gamma }}=K_{PL}\cdot {\dot{\gamma }}^{n-1}$$

(3) $$\sigma =\sigma_{y}+K_{HB}\cdot {\dot{\gamma }}^{m}.$$

In these equations, *n* is the power law flow index, *m* is the Herschel–Bulkley flow index, $\sigma_{y}$ is the yield stress, $K_{PL}$ is the power law consistency index, and $K_{HB}$ is the Herschel–Bulkley consistency index.

### 4.3. Preparation of Hydrogel Formulations

All components of the hydrogel formulations were measured gravimetrically. All hydrogel formulations contained between 17.5% and 22.5% (*w/w*) Poloxamer 407, 0.1% (*w/w*) Irgacure 2959, and 20% (*w/w*) PEG-DA/charged monomer mixture. The rest of the formulations consisted of water, resulting in water contents between 62.4% and 57.4% (*w/w*). The PEG-DA/charged monomer mixture contained amounts of charged monomers such that the final formulations contained 1% (*w/w*), 3% (*w/w*), or 5% (*w/w*) charged monomer (either SPAK or AETA). In order to reach the desired concentrations, first, a stock solution of Irgacure 2959 in water (7 mg/g *w/w*) was prepared by heating to approximately 100 ${}^{\circ }$C with a heat gun and subsequent cooling to room temperature. For the 1 g hydrogel formulation, 143 mg Irgacure 2959 stock solution (containing 1 mg of Irgacure 2959) was added to 200 mg PEG-DA/charged monomer mixture, and an appropriate amount of Poloxamer 407 was added followed by water (e.g., 200 mg Poloxamer 407 and 457 mg water for a Poloxamer 407 concentration of 20% (*w/w*)). The mixture was then left on a roll mixer at 4 ${}^{\circ }$C until a homogenous solution was obtained (approximately 5 days). For all compositions of the tested hydrogel formulations, see Table 1 and Table 2.

### 4.4. Preparation and Characterization of Chemical Hydrogels

Seven hunded and fifty microliters of a hydrogel formulation were pipetted into a cylindrical aluminium mold (diameter 3 cm, height 1 mm) and covered with a quartz glass pane. Chemical curing of hydrogels was achieved by UV curing of the hydrogel formulations in the UV chamber at an irradiation intensity of 50 mW cm^−2^ for 7.5 min. The hydrogels were then transferred into a polystyrene petri dish and washed three times with 20 mL of water, with each washing step taking 24 h. The washing waters were freeze-dried and the residue was analyzed further by gravimetry, ^1^H NMR spectroscopy, and SEC. The equilibrium degree of swelling (*EDS*) of the washed hydrogels was determined using the mass ($m_{swollen}$) of the swollen hydrogel and the mass ($m_{dry}$) of the dried hydrogel by the following equation:

(4) $$EDS=\frac{m_{swollen}}{m_{dry}}\cdot 100\%.$$

Hydrogels were dried in a vacuum chamber at a pressure of 30 mbar and a temperature of 60 ${}^{\circ }$C until a constant mass was achieved (usually 24 h). Dried hydrogels were used for FT-IR spectroscopy. For rheology, hydrogels with a diameter of 2 cm were punched out of the washed hydrogels.

### 4.5. File Preparation and Device Setup for Extrusion-Based 3D Printing

**Figure 9:**
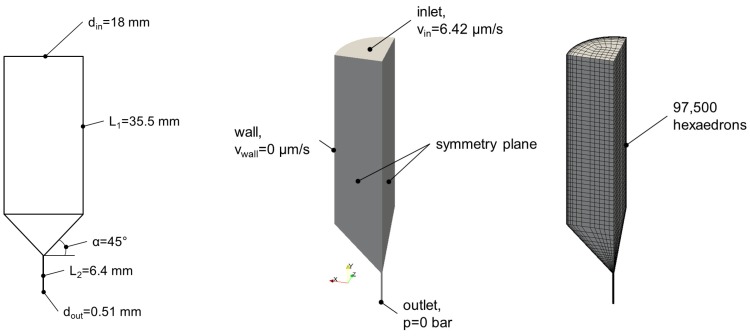
**Left**: Longitudinal section of the printer cartridge used. The cross-section of the dispensing capillary was circular. **Middle**: Parameters used for calculations. **Right**: Mesh used for calculations.

All objects were drawn in the CAD program FreeCAD and encoded for extrusion-based 3D printing using the software Slic3r (http://slic3r.org/) with a layer height of 0.204 mm, making up 40% of the nozzle diameter. Dispensing was performed with a Hyrel 3D System 30 device with dispensing speeds of 8 mm s^−1^ using a nozzle with a circular cross-section with an inner diameter of 0.51 mm. The length of the dispensing capillary was 6.4 mm (for details see also Figure 9). The 3D printing device was operated using the software Repetrel. The dispensing device was equipped with one piston-driven cartridge holding up to 25 mL of ink. Photographs of each dispensed object were taken immediately after dispensing was complete using a Canon EOS 400D camera with a Sigma Aspherical Macro Lens.

### 4.6. Calculation of Flow Profiles during Extrusion-Based 3D Printing

The determination of the flow field inside the nozzle was performed applying numerical methods by means of the open source simulation software package OpenFOAM^®^ in accordance with the Herschel–Bulkley model. The finite volume method was used for discretization of the conservation equations for mass and momentum. The energy equation was neglected assuming isothermal behavior inside the nozzle. The model parameters from Table 2 were used. In Figure 9 (left), the geometry of the printer cartridge and nozzle are shown.

The applied boundary conditions and the mesh can be found in Figure 9 (middle and right). The inlet velocity was determined by the extrusion speed. The velocity at the wall was set to zero with the assumption that no wall slip effects occurred. Due to the incompressible behavior of the hydrogels at the expected pressures, it was assumed that the absolute pressure would not influence the simulation results. Therefore, the pressure at the outlet was also set to zero. Furthermore, symmetry planes were applied to reduce the calculation time.

Overall 97,500 hexaedrons were generated to achieve accurate results within the numerical calculations. A mesh study was performed before to ensure the independency of the simulation results from the applied mesh.

Consequently, the system was completely defined and simulations were performed for all hydrogel formulations from Table 2.

## 5. Conclusions

In this contribution, it was shown that hydrogel formulations suitable for extrusion-based 3D printing containing P407, PEG-DA and negatively and positively charged monomers are available. All hydrogel formulations tested could be transferred from a sol state into a gel state above a certain temperature and showed high shear thinning above this temperature. Formulations containing high $c_{Polox}$ additionally exhibited a relevant yield stress. Although usability of the formulations, e.g., for direct cell encapsulation, is probably limited due to cytotoxicity of P407, the cured and washed hydrogels could be used as scaffolds in tissue engineering or as mineralization templates. Especially for the latter purpose, possible voids resulting from removed P407 micelles together with an enrichment of certain ions inside the hydrogels due to the charged groups might have an interesting effect. Concerning 3D printing, the hydrogel formulation flow properties led to flow profiles in the dispensing needle during 3D printing which were distinctively plug-like, leading to very low shear stress in the majority of the printed volumes and to a thin lubricating layer of highly sheared material very close to the needle wall. Thus, smooth hydrogel strands were formed during printing and 3D objects with defined shapes could be fabricated. Therefore, it was concluded that for the successful extrusion-based 3D printing of hydrogels, the formulation of printed materials in such a way that they have a relevant yield stress and are highly shear thinning is an excellent possibility. However, so far, it remains unclear as to whether there is a practical upper limit for the yield stress. From a theoretical point of view, the flow will become more and more plug-like with increasing yield stress, resulting in a lower volume fraction of the material which is sheared to a relevant extent, thus reducing the importance of fast recovery kinetics of viscosity after shearing. Thus, a high yield stress might open the way to using formulations with a slow viscosity recovery which might improve the connectivity between strands and layers. However, it has been recognized that yield stress can make it difficult to suspend biological cells into formulations [24]. In order to derive generally applicable formulation criteria for hydrogels suitable for extrusion-based 3D printing, a greater number of systematic rheological studies with different materials is necessary. Furthermore, detailed mechanical investigations dealing with the connectivity of strands could clarify the optimum property window which will be defined by interactions of shear thinning, yield stress, viscosity recovery kinetics, and possibly other properties which have not been taken into account yet.

## Figures and Tables

**Table 1:** Composition, flow index (*n*), and consistency index ($K_{PL}$) of the hydrogel formulations containing SPAK or AETA with $c_{Polox}$ = 17.5% obtained from the viscosity curves using Equation (2) (see Section 4.2).

$c_{\mathrm{Polox}}$ [%, *w/w*]	$c_{\mathrm{SPAK}}$ [%, *w/w*]	$c_{\mathrm{AETA}}$ [%, *w/w*]	*n*	log$K_{\mathrm{PL}}$
17.5	1.0	-	0.09	2.09
17.5	5.0	-	0.08	1.99
17.5	-	1.0	0.10	2.05
17.5	-	5.0	0.08	1.92

**Table 2:** Composition, flow index (*m*), consistency index ($K_{HB}$) and yield stress ($\sigma_{y}$) of the hydrogel formulations containing SPAK or AETA with $c_{Polox}$ ≥ 20% obtained from the shear stress curves using Equation (3) (see Section 4.2).

$c_{\mathrm{Polox}}$ [%, *w/w*]	$c_{\mathrm{SPAK}}$ [%, *w/w*]	$c_{\mathrm{AETA}}$ [%, *w/w*]	*m*	log$K_{\mathrm{HB}}$	$\sigma_{y}$ [Pa]
20.0	3.0	-	0.25	1.73	110
22.5	1.0	-	0.31	1.72	160
22.5	5.0	-	0.28	1.76	152
20.0	-	3.0	0.24	1.76	99
22.5	-	1.0	0.28	1.78	156
22.5	-	5.0	0.27	1.80	130

## Supplementary Materials

The following are available at http://www.mdpi.com/2310-2861/4/3/69/s1, Figure S1: Storage moduli G′ and loss moduli G″ of hydrogel formulations containing SPAK (left) and AETA (right) measured 10 ${}^{\circ }$C above the gel transition temperature. The surface fits to the data are for the guidance of the eye only; Figure S2: Recovered yield of Poloxamer 407 upon washing of the hydrogels; Figure S3: Temperature-dependent rheological measurements of all hydrogel formulations used in this study; Figure S4: Calculated shear stress profiles of the different Herschel-Bulkley liquids from the manuscript which have a $c_{Polox}$ ≥ 20%; Figure S5: Viscosity recovery of hydrogel formulations containing AETA in an experiment with sudden changes between low shear rates (0.01 s${}^{-1}$) and high shear rates (300 s${}^{-1}$); Figure S6: Viscosity recovery of hydrogel formulations containing SPAK in an experiment with sudden changes between low shear rates (0.01 s${}^{-1}$) and high shear rates (300 s${}^{-1}$).
