# Supplementary material for: Extrusion-Based 3D Printing of Poly(ethylene glycol) Diacrylate Hydrogels Containing Positively and Negatively Charged Groups
Source: Gels. 2018 Aug 14;4(3):69. doi: 10.3390/gels4030069 (PMC6209279; doi:10.3390/gels4030069)
Supplement: Supplementary file 1 [file gels-04-00069-s001.pdf]

Supporting Information for

Extrusion-based 3D printing of poly(ethylene glycol) diacrylate hydrogels containing positively and negatively charged groups

Sebastian Joas, Günter E.M. Tovar, Oguz Celik, Christian Bonten, Alexander Southan

Table of contents

1. Storage and loss moduli of physical hydrogels 2
2. Recovered yield of Poloxamer 407 upon hydrogel washing 2
3. Temperature-dependent rheology of hydrogel formulations 3
4. Shear stress during 3D printing according to the Herschel-Bulkley model 4
5. Viscosity recovery of hydrogel formulations after shearing 4

1. Storage and loss moduli of physical hydrogels

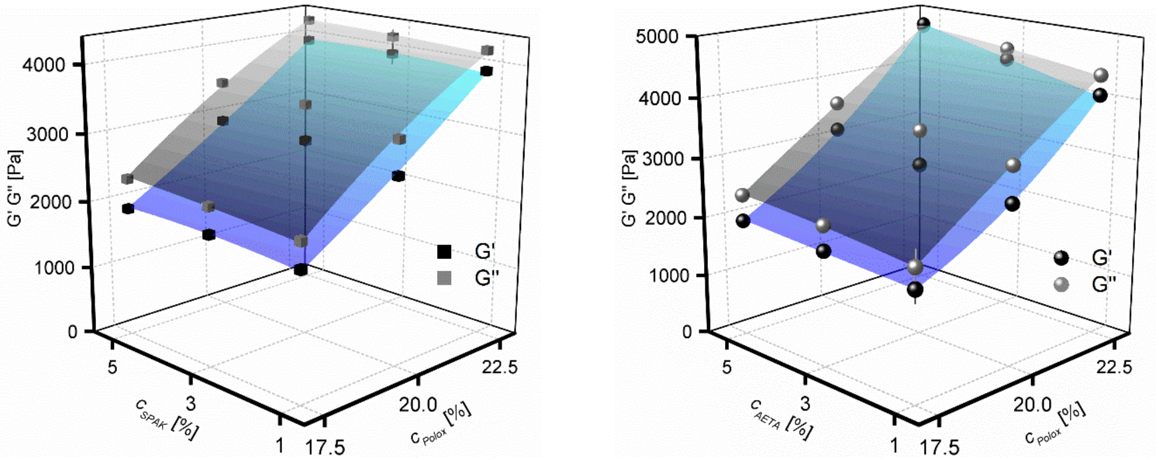

FIGURE S1 Storage moduli G‘ and loss moduli G“ of hydrogel formulations containing SPAK (left) and AETA (right) measured 10 °C above the gel transition temperature. The surface fits to the data are for the guidance of the eye only.

2. Recovered yield of Poloxamer 407 upon hydrogel washing

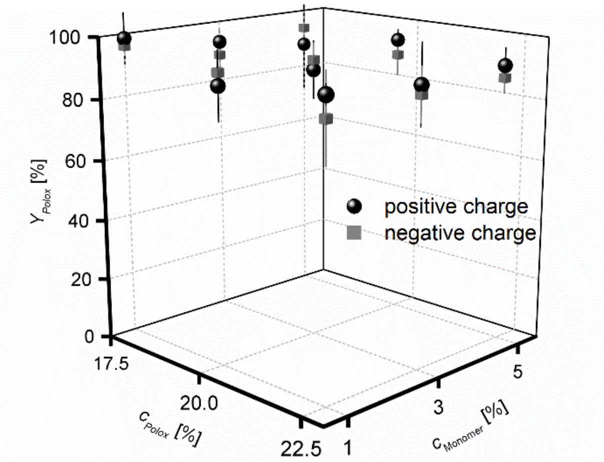

FIGURE S2 Recovered yield of Poloxamer 407 upon washing of the hydrogels.

3. Temperature-dependent rheology of hydrogel formulations

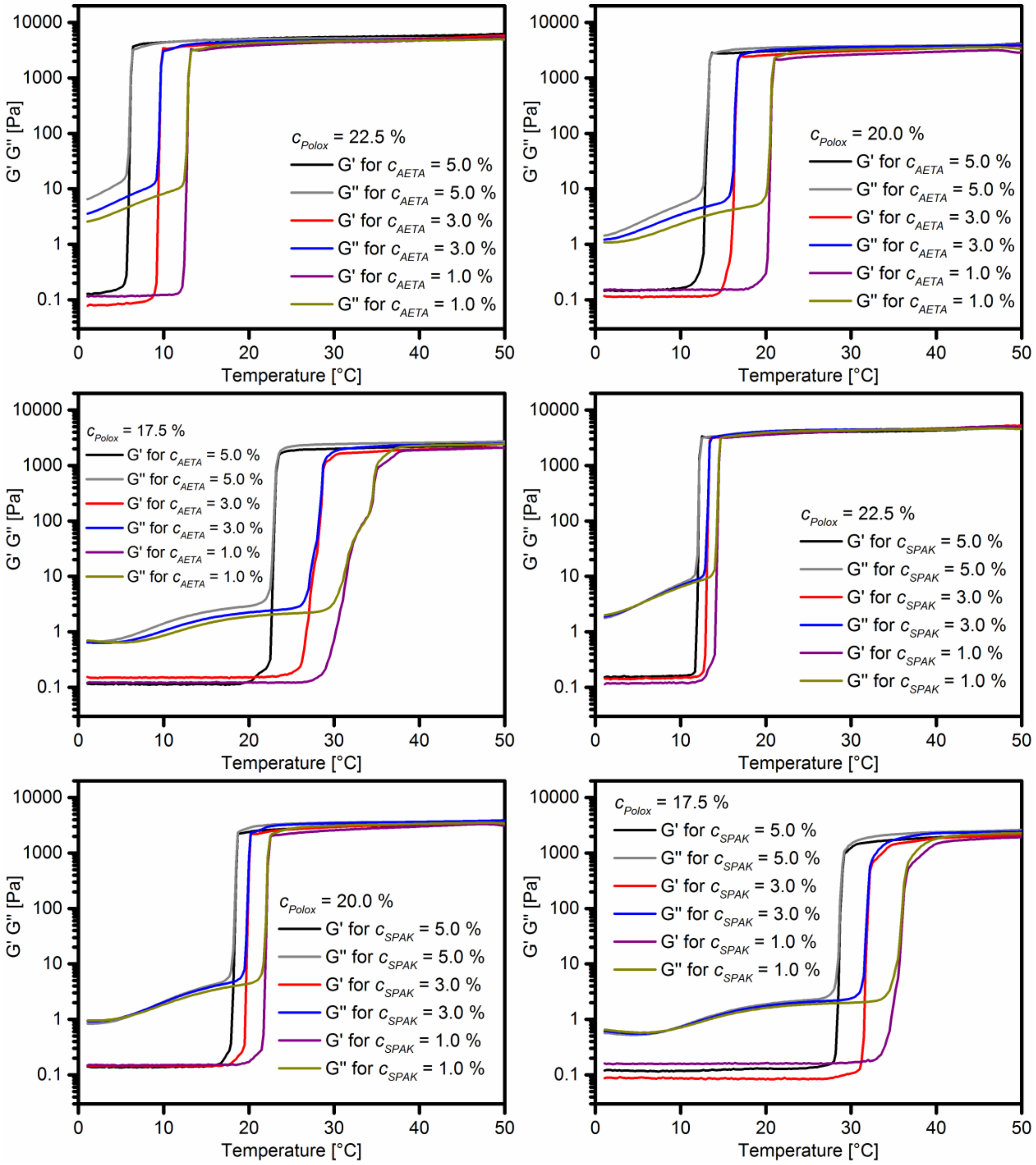

FIGURE S3 Temperature-dependent rheological measurements of all hydrogel formulations used in this study.

4. Shear stress during 3D printing according to the Herschel-Bulkley model

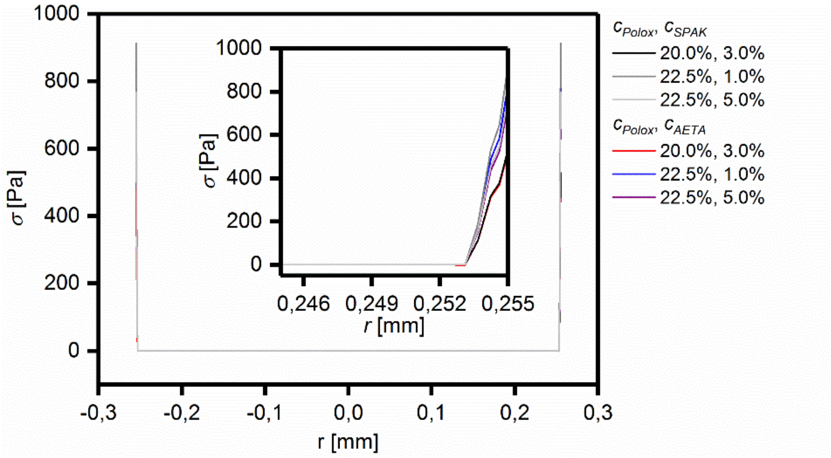

FIGURE S4 Calculated shear stress profiles of the different Herschel-Bulkley liquids from the manuscript which have a $c_{Polox} \geq 20\%$.

5. Viscosity recovery of hydrogel formulations after shearing

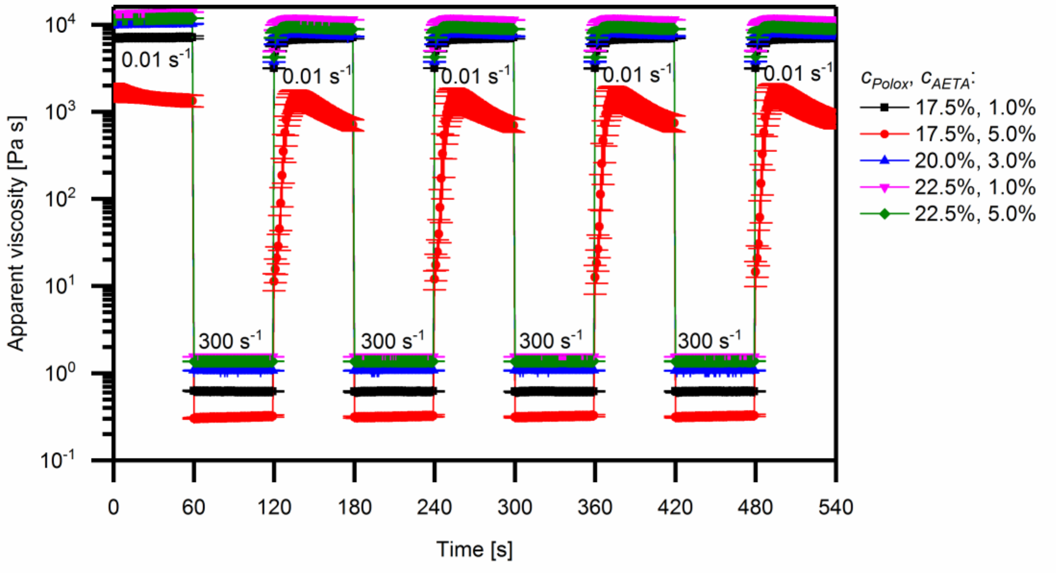

FIGURE S5 Viscosity recovery of hydrogel formulations containing AETA in an experiment with sudden changes between low shear rates (0.01 s^{-1}) and high shear rates (300 s^{-1}).

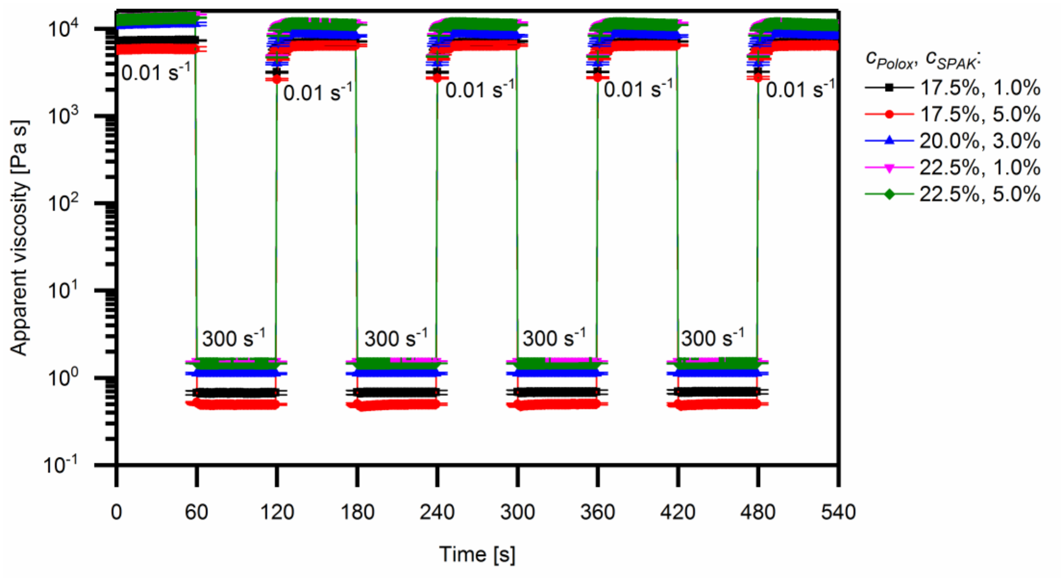

FIGURE S6 Viscosity recovery of hydrogel formulations containing SPAK in an experiment with sudden changes between low shear rates (0.01 s^{-1}) and high shear rates (300 s^{-1}).
